# *Anisakis pegreffii* Extract Induces Airway Inflammation with Airway Remodeling in a Murine Model System

**DOI:** 10.1155/2021/2522305

**Published:** 2021-09-17

**Authors:** Jun Ho Choi, Ju Yeong Kim, Myung-hee Yi, Myungjun Kim, Tai-Soon Yong

**Affiliations:** Department of Environmental Medical Biology, Institute of Tropical Medicine & Arthropods of Medical Importance Resource Bank, Yonsei University College of Medicine, Seoul 03722, Republic of Korea

## Abstract

Exposure of the respiratory system to the *Anisakis pegreffii* L3 crude extract (AE) induces airway inflammation; however, the mechanism underlying this inflammatory response remains unknown. AE contains allergens that promote allergic inflammation; exposure to AE may potentially lead to asthma. In this study, we aimed to establish a murine model to assess the effects of AE on characteristic features of chronic asthma, including airway hypersensitivity (AHR), airway inflammation, and airway remodeling. Mice were sensitized for five consecutive days each week for 4 weeks. AHR, lung inflammation, and airway remodeling were evaluated 24 h after the last exposure. Lung inflammation and airway remodeling were assessed from the bronchoalveolar lavage fluid (BALF). To confirm the immune response in the lungs, changes in gene expression in the lung tissue were assessed with reverse transcription-quantitative PCR. The levels of IgE, IgG1, and IgG2a in blood and cytokine levels in the BALF, splenocyte, and lung lymph node (LLN) culture supernatant were measured with ELISA. An increase in AHR was prominently observed in AE-exposed mice. Epithelial proliferation and infiltration of inflammatory cells were observed in the BALF and lung tissue sections. Collagen deposition was detected in lung tissues. AE exposure increased *IL-4*, *IL-5*, and *IL-13* expression in the lung, as well as the levels of antibodies specific to AE. IL-4, IL-5, and IL-13 were upregulated only in LLN. These findings indicate that an increase in IL-4^+^ CD4^+^ T cells in the LLN and splenocyte resulted in increased Th2 response to AE exposure. Exposure of the respiratory system to AE resulted in an increased allergen-induced Th2 inflammatory response and AHR through accumulation of inflammatory and IL-4^+^ CD4^+^ T cells and collagen deposition. It was confirmed that *A. pegreffii* plays an essential role in causing asthma in mouse models and has the potential to cause similar effects in humans.

## 1. Introduction

The fish-borne zoonotic parasites *Anisakis simplex* and *A. pegreffii* are known to cause anisakiasis or allergies in humans [1]. The estimated frequency of such reactions is 200 cases per year in South Korea, 2000 cases in Japan, and 20–500 cases in some European countries [2, 3]. A notable sign of *Anisakis* allergy is a reaction that occurs when live larvae penetrate the gastric mucosa, which is commonly associated with hives, angioedema, abdominal pain, and irritability [4, 5]. The gastrointestinal symptoms may be minimal or absent, and the onset of symptoms is usually delayed between 2 and 24 h [2]. This delay between consumption of fish and the onset of symptoms can be an important diagnostic clue for the detection of *Anisakis* allergy. Workers involved in the manual or automated processing of crabs, shrimps, mussels, fishes, and fishmeal are typically exposed to various seafood ingredients [6]. Aerosolization of seafood and cooking fluids during processing is a potential occupational hazard that can cause sensitization through inhalation [6]. Indeed, allergic and anaphylactic reactions to *Anisakis* have been reported among workers in fish processing plants [7, 8]. *A. pegreffii* and *A. simplex* allergens are known to contribute to respiratory allergies and contact dermatitis [9, 10]; repeated inhalation of the aerosolized anisakid protein may trigger a respiratory reaction, as suggested by prior studies in humans [6, 11]. A case of occupational hypersensitivity to *Anisakis* had previously been reported in a worker in a frozen fish factory. Systemic hives, rash, and symptoms of asthma were observed in the worker after exposure in the workplace. All the symptoms disappeared immediately after workplace exposure ceased [12]. This indicates that *Anisakis* is a significant cause of occupational asthma and hives in the fish industry [12]; additionally, anaphylactic reactions due to *Anisakis* have been confirmed in animal models [13]. Cases of asthma have also been observed from exposure to *Anisakis* [12, 14, 15].

A major allergen (Ani s 7) has been identified in *Anisakis* species [16, 17]. Additionally, proteolytic enzymes in the body of whale roundworms are well known as allergens [16]. Several factors may contribute to the increased antigenicity and allergenicity of Ani s 7, including the repeating amino acid composition of the motif, high cysteine content, and the ability to create allergen resistance to proteolysis by certain prominent enzymes (i.e., cathepsin) in the intracellular pathway of antigen-presenting cells by specific T cells [18]. However, it has been observed that skin tests using whole body extracts of parasite larvae or oral administration of freeze-dried parasites do not reproduce clinical symptoms. Hence, the secretions or secreted proteins produced by live larvae have been suggested as possible allergens [19, 20]. Furthermore, in an *A. simplex* allergy study, it was demonstrated that cooking and freezing did not destroy allergens and parasitic substances and could not protect against hypersensitivity reactions [8, 21, 22]. Thus, these allergens are resistant to the external environment and can sustainably induce allergic reactions upon exposure.

Asthma is a common inflammatory airway disease that affects over 300 million people worldwide [23]. It has increasingly been recognized as a heterogeneous disease with various characteristics, particularly in chronic cases of the disease [24, 25]. Airway remodeling is a prominent feature of asthma that can occur prior to the onset of symptoms [26–28]. When airway remodeling occurs, changes in collagen deposition, degree of fibroblast/myofibroblast accumulation, degree of airway smooth muscle (ASM) volume, and decreased distance from the epithelium to ASM are the only morphological features that distinguish persistent asthma from mild disease. Hence, airway remodeling is an essential phenomenon even in animal models of asthma. Several mouse models of allergic airway inflammation have been established to investigate various features of asthma [7, 29–32]. Acute airway inflammation models, usually induced within 3 weeks, are often characterized by airway hypersensitivity (AHR) and inflammation, but not remodeling [33, 34]. Notably, in a 1-week model system using *Anisakis* crude extract allergens, the number of cells involved in the inflammatory immune response increased; however, observations of airway remodeling, indicative of asthma, were not reported [35]. Airway remodeling features, such as goblet cell proliferation, were not observed in the 2-week model as well [33]. Indeed, airway remodeling through collagen deposition is usually not observed in mouse models until mice are exposed to allergens for more than 4 weeks [36, 37]. Interestingly, airway remodeling has not been reported in the 6- and 10-week chronic *Anisakis* asthma model systems [13].

In this study, we established a 4-week mouse model of *Anisakis*-induced allergic airway inflammation using daily intranasal treatment with the *A. pegreffii* extract (AE). The airway remodeling markers such as collagen deposition as well as general allergic asthma markers were assessed. We propose that this 4-week experimental murine model with AE exposure may be suitable for studying chronic asthma with allergic airway remodeling.

## 2. Materials and Methods

### 2.1. Animals

Female BALB/c mice (n = 10; 8 weeks old) were purchased from Orient Bio (Seongnam, South Korea). Of the 10 mice, 5 were included in the control group, and the remaining 5 were included in the experimental group. All animal studies were approved by the Department of Laboratory Animal Resources Committee of Yonsei University College of Medicine (no. 2018-0316, 2020-0077). The mice were housed in specific pathogen-free conditions and a 12 h light/dark cycle and acclimated for a week before the start of the experiment. The health of the animals was monitored daily.

### 2.2. Allergen Extraction

Chub mackerel (*Scomber japonicus*) were purchased from a traditional market in Seoul, South Korea. *A. pegreffii* third-stage larvae (L3) were manually harvested from the abdominal cavity. The genotypes of *A. pegreffii* and anisakid larvae were confirmed with polymerase chain reaction (PCR) using the protocol described by Lee et al. The primers used were ASF1 5′-CAG CTT AAG GCA GAG TC-3′ and AS2 5′-TAT CAT TTT TGA TCA CAT AGA C-3′ [38].

*A. pegreffii* were washed with distilled water and then stored at 4°C. Then, AE was prepared by washing the L3 larvae with sterile water, followed by sonication. The extract was centrifuged for 30 min at 10,000 × g and filter-sterilized through a 0.22 *μ*m filter (Millipore, Seoul, South Korea). Protein concentration was measured using the Bradford test (Bio-Rad, Hercules, CA, USA) according to the manufacturer's instructions. The extract was kept on ice during the extraction process and stored at −80°C until required. The AE was used for sensitization, as well as exposure challenge and enzyme-linked immunosorbent assay (ELISA) experiments.

### 2.3. Occurrence of Allergic Airway Inflammation

**Figure 1:**
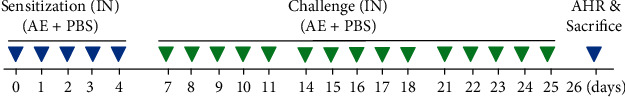
Timeline for allergen exposure in a chronic 4-week *Anisakis pegreffii*-treated mouse model system. Mice were treated with 25 *μ*g of the *A. pegreffii* extract (AE) in 35 *μ*L phosphate-buffered saline (PBS) or 35 *μ*L PBS alone by an intranasal challenge for five consecutive days in a week for a period of 4 weeks.

The AE (25 *μ*g) was resuspended in phosphate-buffered saline (PBS), and 35 *μ*L AE solution was inoculated into the mice intranasally using a pipette tip (Figure 1). The control mice received 35 *μ*L of sterile PBS. The mice were sensitized for five consecutive days each week (Monday–Friday) for 4 weeks. The endpoint metrics were assessed 24 h after the last AE or PBS exposure.

### 2.4. Assessment of Lung Function and Methacholine Reactivity

AHR was measured 24 h after the last treatment, using the FlexiVent system (Scireq Inc., Montreal, QC, Canada) as previously described [39]. Briefly, mice were anesthetized with ketamine (36 mg/kg IP; Zoletil® 50, Virbac, South Korea) and xylazine (11.2 mg/kg IP; Rompun inj, Bayer, South Korea). An intratracheal 18-gauge stainless steel cannula (LS18, Luer stub, green ×0.5 in 12 mm, nonsterile, Instech, Plymouth Meeting, PA, USA) was used for intubation to measure respiratory dynamics, along with a ventilator-based FlexiVent® system. Pancuronium bromide (0.8–1.2 mg/kg IP; P1918 Sigma-Aldrich, St. Louis, MO, USA) was administered prior to the lung function tests to prevent self-breathing. Mice were subjected to deep lung inflation, which involved slow inflation of the lung with a pressure of 27 cm H_2_O maintained for 6 s, followed by one time delivery of 0.1 ml of 3 cm H_2_O applied with a water trap for positive end-expiratory pressure (PEEP). Ventilation was performed at 150 breaths/min with a respiratory volume of 10 mL/kg. The baseline measurement of respiratory dynamics (quasi-static compliance) increased the concentration of normal nebulized PBS (used for baseline measurement) and methacholine (MCh; Sigma-Aldrich) (untreated BALB/c, n = 5). AHR was measured in antigen-sensitive and challenged BALB/c (n = 5) mice under three consecutive concentrations of MCh (12.5, 25, and 50 mg/mL). A 3 s broadband with 2 s perturbation, 2.5 Hz single-frequency forced oscillation technique maneuver (SFOT; using SnapShot-150 perturbation), and 12 mutual prime frequencies between 1 and 20.5 Hz at 5 s intervals for a total of 3 min were applied. These were evaluated using the low-frequency forced oscillation technique maneuver (LFOT; using Quick Prime-3 perturbation).

The airway constriction (respiratory resistance system (Rrs)) and airway stiffness (elastance of the respiratory (Ers)) were calculated using the FlexiVent software by fitting the equation of motion of a linear single-compartment model of lung mechanics to SFOT data using multiple linear regression. Respiratory system input impedance was calculated from the LFOT data, and central airways (Newtonian resistance (Rn)) and tissue mechanics (alveolar tissue damping (G) and alveolar tissue elastance (H)) were determined by the input impedance. Both FOT maneuvers were run alternately every 5 s after each MCh aerosol challenge to capture the time course and determine the detailed bronchoconstriction response after MCh treatment. Assessment of allergen-induced AHR by FOT was performed using a 10 s spray period synchronized with inspiration at a spray rate of 50% after DI, and baseline measurements were made to deliver a PBS solution to the mice as an aerosol. The FOT measurement was used to monitor the time course of the subsequent response as described above.

### 2.5. Bronchoalveolar Lavage Fluid (BALF) Collection and Processing

In order to collect BALF, mouse lungs were irrigated using tracheal tubes with 1 mL Hank's balanced salt solution (HBSS, Thermo Fisher Scientific, Waltham, MA, USA), and total cell counts were determined using a hemocytometer. The collected BALF samples were centrifuged for 5 min at 3,000 × g and 4°C. The whole-cell pellet was resuspended in HBSS, and BALF cell smears were prepared by cell centrifugation (Shandon Cytospin 4, Thermo, Seoul, South Korea). The slides with cell smears were stained with Diff-Quik (Sysmex, Tokyo, Japan) and analyzed using previously described methods [40].

### 2.6. Histological Analysis

For mouse lung tissue analysis, the left lung from each mouse was isolated, fixed in 10% formalin for 24 h, and embedded in paraffin. The lung tissue samples were sectioned (2–3 *μ*m) and stained with hematoxylin and eosin (H&E) and periodic acid-Schiff (PAS) using standard histological protocols to detect mucus-containing cells [41]. Goblet cell hyperplasia was measured, and fibrosis was assessed, as previously described, using Masson's trichrome stain [42]. Tissue sections were examined using an Olympus BX53 microscope with an Olympus DP71 digital camera (magnification, 400x; Olympus DP71, Nishi Shinjuku 2-Chome, Tokyo, Japan). Images were acquired using the cellSens standard 1.12 imaging software (Olympus, Tokyo, Japan).

The pathological change index of H&E slides was assigned numerical values based on inflammatory cell infiltration and thickness around the airway and blood vessels (0, normal or no cells; 1, ≤3 cell diameter thickness; 2, 4–6 cell thickness; 3, 7–9 cell thickness; and 4, ≥10 cell thickness). Similarly, numerical values were assigned according to the proportion of airways and blood vessels in each section that were surrounded by inflammatory cells (0, normal or no airways or blood vessels; 1, <25% of the airways or blood vessels; 2, 25–50%; 3, 51–75%; and 4, ≥75%). The exponent was calculated by multiplying the severity by the range, with a maximum possible score of 9. The number of mucus-containing cells/mm^2^ of the basement membrane and bronchial and perivascular inflammation intensity was also measured. Furthermore, airway epithelial cells were scored on the degree of goblet cell hyperplasia, on a scale of 0–3 (0 = no inflammation, goblet cell metaplasia, or <75% of PAS^+^ cytoplasm, 1 = mild inflammation, goblet cell metaplasia, or >75% of PAS^+^ cytoplasm, 2 = moderate inflammation or goblet cell metaplasia, and 3 = strong inflammation or goblet cell metaplasia). PAS^+^ cells in the epithelial region were counted six times per section in two tissue sections per mouse (n = 5 mice/group) [43–48]. Each value is expressed as mean ± SD.

### 2.7. Total RNA Extraction and Reverse Transcription-Quantitative PCR (RT-qPCR)

The lungs of mice were harvested, suspended in 1 mL of RNAlater® (Life Technologies, Burlington, ON, Canada), and stored at −20°C. Two micrograms of total RNA was extracted from the lungs using 1 mL of RiboEx™ (301-001; Seoul, South Korea), and cDNA was synthesized using MMLV reverse transcriptase (ENZ-KIT106-0200; Ampigene® cDNA Synthesis Kit, Farmingdale, NY, USA) according to the manufacturer's instructions. Gene expression profiling was performed for the following targets: *IL-4*, *eotaxin-1* (*chemokine*, *C-C motif ligand 11*, and *Ccl11*) [43], *IL-5* [44], *IL-13* [45], *IL-17A*, and *Cxcl1* [43]. The target genes were quantified and analyzed using the qPCR Green Mix Hi-ROX kit (ENZ-NUC104-1000, Ampigene®, Enzo Life Sciences, Farmingdale, NY, USA) according to the manufacturer's instructions. The reaction was performed on a real-time PCR machine (StepOne Plus, Applied Biosystems Inc., Seoul, South Korea) using the primer sequences listed in Supplementary Table 1. The relative expression of each gene was calculated as the ratio of target gene expression to the housekeeping gene peptidylprolyl isomerase A (*Ppia*) expression, using the StepOne software (v2.3, Applied Biosystems Inc.).

### 2.8. Measurement of Immunoglobulin (Ig) and Cytokine Levels

After the lung function test was completed, mice were euthanized with an excess of ketamine/xylazine and blood was collected through the abdominal vein [46]. The serum levels of *A. pegreffii*-specific IgE were evaluated using ELISA. Briefly, 96-well plates (Corning® 1 × 8 Stripwell™ 96-well plates, Sigma-Aldrich Co., Seoul, South Korea) were coated with 0.5 *μ*g AE in 50 *μ*L coating buffer and incubated overnight at 4°C. The plates were blocked with 100 *μ*L/well of blocking buffer (1% BSA in PBS). A 50 *μ*L aliquot of the serum sample (0.05% Tween-20 and 0.1% BSA in PBS diluted to 1 : 4) was added to each well and incubated overnight at 4°C. The wells were then washed with wash buffer (0.05% Tween-20 in PBS) and incubated with the appropriate antibodies (50 *μ*L) for 2 h. The antibodies included biotinylated anti-mouse IgE (1 : 1000; 408804; BioLegend, San Diego, CA, USA), biotinylated goat anti-mouse IgG1 secondary antibody (1 : 10000; NBP1-69914B; Novus Biologicals, Littleton, CO, USA), and biotin goat anti-mouse IgG2a secondary antibody (1 : 10000; NBP1-69915B; Novus Biologicals). The wells were then incubated with an avidin-horseradish peroxidase (HRP) conjugate (BioLegend) for 30 min, followed by incubation with the 3,3′,5,5′ tetramethyl benzidine (TMB) substrate (50 *μ*L) in the dark for 5 min. The reaction was stopped with 2 N·H_2_SO_4_ (50 *μ*L). The absorbance was assessed at 450 nm using VersaMax (Molecular Devices, Seoul, South Korea). Data were normalized to each PBS exposure control.

The splenocytes were isolated to analyze cytokine levels and for cell culture. The isolated splenocytes and lung lymph nodes (LLN) were treated with an ACK hypotonic lysis solution (Sigma-Aldrich) for lysis of erythrocytes for 2 min at room temperature (25°C). After erythrocyte lysis, the remaining cells were filtered through a 100 *μ*m mesh (Small Parts Inc. Miramar, FL, USA) and suspended in RPMI 1640 containing 10% (*v*/*v*) heat-inactivated FBS (HyClone, Logan, UT, USA). The cells (5 × 10^6^ cells/mL) were then plated on 48-well plates (30024, SPL, Gyeonggi-do, South Korea) in RPMI 1640 media containing 100 U/mL penicillin and 100 *μ*g/mL streptomycin (LS202-02, Welgene, Gyeongsan-si, South Korea).

For CD3 stimulation experiments, the cells were treated with 0.5 *μ*g/mL of the CD3e monoclonal antibody (MA5-17655, 145-2C11, Invitrogen, Seoul, South Korea). The plated cells were incubated for 72 h at 37°C and 5% CO_2_. After incubation, the culture medium was harvested and stored at −20°C. The levels of IL-4, IL-5, IL-6, IL-13, and IFN-*γ* were measured using ELISA (PeproTech, Cranbury, NJ, USA). The BALF supernatant and culture supernatant of splenocytes and LLN were assessed using an ELISA kit (PeproTech) according to the manufacturer's instructions. The absorbance of the final reaction was measured at 450 nm as previously described.

### 2.9. Flow Cytometry

To assess the recruitment of IL-4^+^ CD4^+^ T cells, live cells were isolated from splenocytes and LLN from allergic airway inflammatory mice that were or were not sensitized to the *A. pegreffii* crude extract. The cell preparation method was the same as that described in Section 2.8. Samples were measured and analyzed on a flow cytometer (3-laser, 10-color; SONY SA3800) using the appropriate mAbs. The antibodies used for cell surface staining included purified rat anti-mouse CD16/CD32 (553142; Mouse BD Fc Block™, BD Pharmingen™), CD4 monoclonal antibody (T helper cell marker, 17-0042-82; RM4-5, APC, eBioscience, San Diego, CA, USA), and rat IgG2a kappa isotype control (17-4321-81; eBR2a, APC, eBioscience), while intracellular staining was performed with PE-Cy™7 rat anti-mouse IL-4 (560699; BD Pharmingen™), PE-Cy™7 rat IgG1*κ* isotype control (557645; BD Pharmingen™), anti-iNOS-PE cyanine7 (25-5920-82), and anti-arginase 1-PerCP-eFluor 710 (46-3697-82; eBioscience). Additionally, the Intracellular Fixation and Permeabilization Buffer (BD Cytofix/Cytoperm Plus Kit with BD GolgiPlug, 555028; BD Pharmingen™) was used. The experiment was set up according to the recommendations of BD Pharmingen. During sample gating, cells were gated against LLN. The LLN gate determined CD4^+^ cells. IL-4^+^ T cell expression was determined from the gated population.

### 2.10. Statistical Analysis

All results are expressed as mean ± SEM. Statistical analysis was performed using the GraphPad Prism 9.0 software (GraphPad, Inc., La Jolla, CA, USA). The graphs were created on Excel 2016 and GraphPad Prism 9.0 software (GraphPad). In the AHR, Ig, and cytokine, histological score, and FACS cell number experiments, binary comparisons were performed using the unpaired *t*-test where appropriate. The AHR multivariate data were evaluated for group differences using repeated analysis of variance measures and one-way or two-way ANOVA followed by Bonferroni's post hoc test as applicable. The remaining data were analyzed with ANOVA followed by Bonferroni's post hoc test. *p* values < 0.05 were considered statistically significant.

## 3. Results

### 3.1. AE Sensitization and Challenge Cause Airway Hyperresponsiveness in the Mouse Model

**Figure 2:**
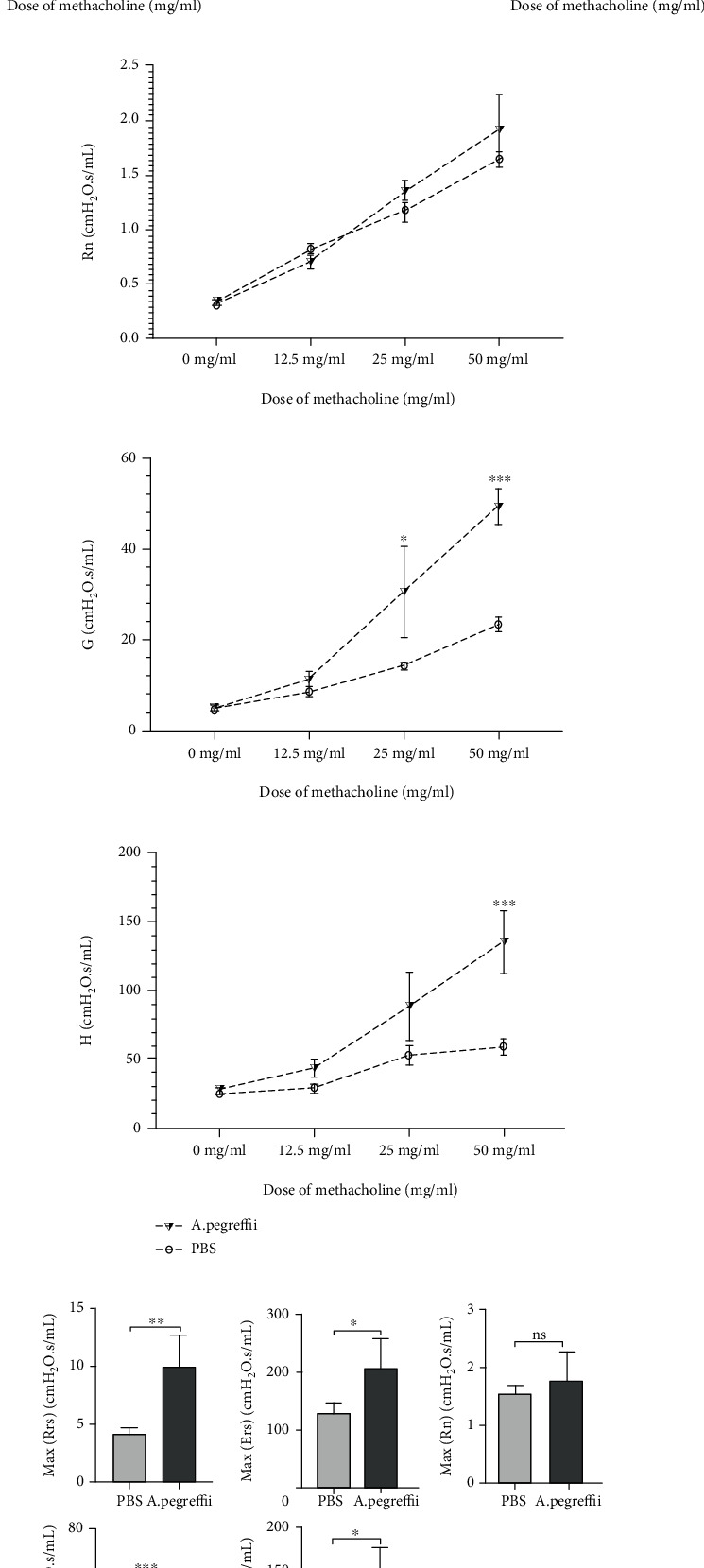
Improved methacholine reaction occurs in the 4-week *Anisakis pegreffii* crude extract model. (a–e) Pulmonary function evaluation was performed by measuring the total respiratory resistance system (Rrs), elastance (Ers), alveolar tissue damping (G), and alveolar tissue elastance (H) in all mice exposed to the *A. pegreffii* extract (AE) compared with the phosphate-buffered saline (PBS) control group. A significant increase in methacholine was observed in H (a–e; ^∗^*p* < 0.05; *n* = 5/group). (f) Except for total (or maximum) Newtonian resistance (Rn) max, the increase in total (or maximum) airway resistance (Rrs max), total (or maximum) airway stiffness (Ers max), total (or maximum) alveolar tissue damping (G max), and total (or maximum) alveolar tissue elastance (H max) was similar in all *A. pegreffii* crude extract groups compared to the PBS control group (f; ^∗^*p* < 0.05; *n* = 5/group).

In order to assess airway function, mice sensitized and challenged with AE for 4 weeks were compared to control mice exposed to PBS. The total Rrs, Ers, G, and H showed enhanced MCh reactivity (Figure 2; PBS at p < 0.05, n = 5/group). Interestingly, mice sensitized and challenged with AE did not significantly increase the Rn values of the central airways.

### 3.2. Measurement of Specific Antibodies in Sera

**Figure 3:**
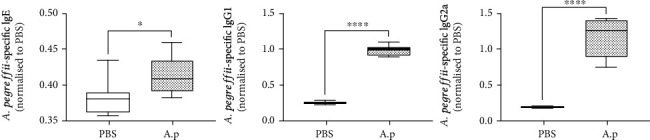
*Anisakis pegreffii* extract (AE) treatment increases the expression of *A. pegreffii*-specific IgE, IgG1, and IgG2a. Measurement of *A. pegreffii*-specific IgE (1 : 1000), IgG1 (1 : 10000), and IgG2a (1 : 10000) levels by ELISA (^∗^*p* < 0.05; ^∗∗^*p* < 0.01; ^∗∗∗^*p* < 0.001; *n* = 5 mice/group).

Serum AE-specific IgE levels were significantly elevated compared with those in the PBS control in the 4-week experimental model system (Figure 3, p < 0.05, PBS vs. AE, n = 5/group). Furthermore, AE-specific IgG1 and IgG2a levels were significantly higher in the AE group than in the PBS treatment group.

### 3.3. Airway Inflammation

**Figure 4:**
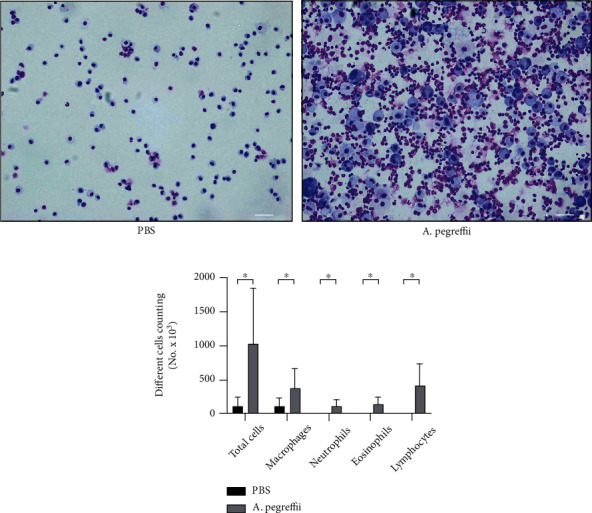
Treatment with the *Anisakis pegreffii* extract (AE) increases the total leukocyte count in the bronchoalveolar lavage fluid and the production of eosinophils. (a, b) Typical micrographs and eosinophil counts in the bronchoalveolar lavage fluid (indicated in blue, pink, and red) of mice challenged with phosphate-buffered saline (PBS) and AE, stained with Diff-Quik, are shown. Original magnification, 200x. Data are presented as mean ± SEM (*n* = 5; ^∗^*p* < 0.05; bar = 100 *μ*m in each group).

In the BALF, total leukocyte count demonstrated a gradual and significant increase over time (Figure 4(a), p < 0.05, *A. pegreffii* vs. PBS, n = 5–7/group). The 4-week AE model showed strong immune cell recruitment to the airways. Differential cell counts revealed an increase in the absolute cell counts of neutrophils, eosinophils, and lymphocytes (Figure 4(b), p < 0.05, *A. pegreffii* vs. PBS, n = 5–7/group). In the 4-week 25 *μ*g AE model system, an increase in eosinophils was consistent with an increase in serum *A. pegreffii*-specific IgE. Monocytes dominated the total BAL leukocyte count, and the absolute cell count was 4.06 × 10^5^ monocytes/mouse and 1.33 × 10^5^ eosinophils/mouse. Absolute BALF eosinophil count was higher than that of the leukocyte subgroup, and the total fold increase was second only to that of monocytes. Compared with that in the PBS control group, the relative increase in eosinophil count in AE mice was 13.1 times. Similarly, the relative increase in neutrophils and lymphocytes in AE mice was 9.4 and 39.5 times, respectively, compared with that in the PBS control group.

### 3.4. Airway Remodeling

**Figure 5:**
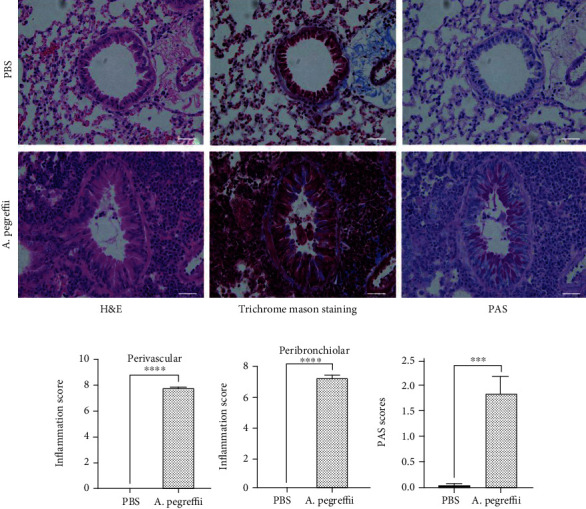
Treatment with the *Anisakis pegreffii* extract (AE) increases inflammatory cell infiltration and mucus production. (a) Sections of the lung were stained with hematoxylin and eosin, periodic acid-Schiff (PAS, goblet cell hyperplasia colored purple-magenta), and Masson's trichrome (collagen deposition colored cyan). Histological appearance of the lungs after treatment with AE (bar = 50 *μ*m). (b) In hematoxylin and eosin staining, some of the swollen lung sections were examined in a blinded fashion under a light microscope to evaluate the “inflammation score” of each section, which is a product of the severity and prevalence of inflammation. The percentage of PAS^+^ cells in the epithelial area was assessed in six sections per mouse. Each value was expressed as mean ± SEM (^∗∗^*p* < 0.01; ^∗∗∗^*p* < 0.001, *n* = 5 mice/group).

After induction of airway inflammation, inflammatory cells were observed in the peribronchial space. Using PAS staining, goblet cell hyperplasia and large amounts of mucus production were detected in the airways of AE-treated mice (Figure 5(a), PAS). Additionally, subepithelial collagen deposition (Figure 5(a), Masson trichrome) was observed (p < 0.05, n = 5/group) in the AE-treated mice. Moreover, after AE treatment, the number of inflammatory cells was significantly higher in the perivascular and peribronchial regions of AE-treated mice than in the control mice (Figure 5(b)). Additionally, the proportion of PAS^+^ cells in the epithelial region of AE-treated mice was more than doubled.

### 3.5. Expression of Inflammatory Mediators

**Figure 6:**
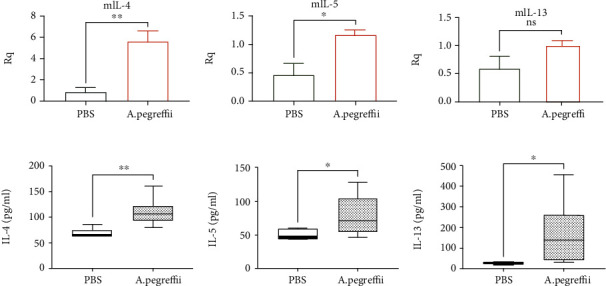
Increased expression of Th2 cytokines in lung lymph nodes (LLN) of the 4-week *Anisakis pegreffii* extract (AE) model. (a–c) Based on the reverse transcription-qPCR analysis of LLN tissue, the expression of *IL-4*, *IL-5*, and *IL-13* was significantly increased in the AE-sensitized model compared with the phosphate-buffered saline (PBS) group (d–f). IL-4, IL-5, and IL-13 (d–f) in the cell culture supernatant of CD3-stimulated lymphocytes isolated from LLN (d–f) showed an elevated expression pattern (^∗^*p* < 0.05; *n* = 5/group).

Assessment of gene expression in the lungs using RT-qPCR revealed a pattern of elevated expression of Th2-type cytokines in the AE mouse model. Expression of the inflammatory mediators *IL-4*, *IL-5*, and *IL-13* was upregulated when exposed to AE (Figures 6(a)–6(c), p < 0.05, AE vs. PBS, n = 5/group). Mice exposed to AE demonstrated no difference in the expression of the strong neutrophil chemoattractants *Cxcl-1* and *IL-17A* compared with the control group; however, *IL-6* expression was found to increase in the AE group (not significant). The eosinophil chemoattractant *eotaxin-1* demonstrated an increase in expression, but no significant difference was observed (Supplementary Figure S1, p < 0.05, AE vs. PBS, n = 5/group).

**Figure 7:**
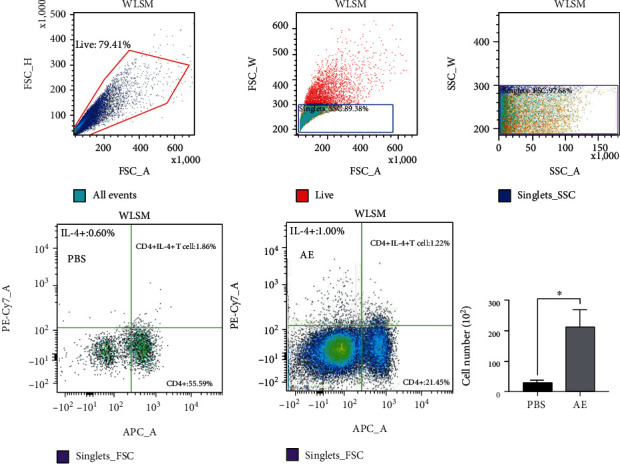
Expression of the IL-4^+^ CD4^+^ marker in lung lymph nodes (LLN). Expression of the IL-4 marker in the LLN of *Anisakis pegreffii* extract- (AE-) sensitized and control mice was analyzed using flow cytometry. Lymphocytes from LLN were incubated with a stimulated anti-CD3e antibody. After staining, lymphocytes were initially gated for CD4^+^ cells, and the percentage of IL-4^+^ cells was calculated using FACS analysis. The IL-4^+^ CD4^+^ T cell number is plotted in the right panel.

An increased Th2 response was observed in the LLN culture supernatant after treatment with AE. The LLN cells from AE-induced mice exhibited significant increase in IL-4, IL-5, and IL-13 cytokine levels in the culture medium of these cells (Figures 6(d)–6(f), p < 0.05, AE vs. PBS, n = 5/group). Additionally, the levels of IFN-*γ*, a cytokine secreted by Th1, were significantly increased in samples obtained from the AE group (Supplementary Figure S1, p < 0.05, AE vs. PBS, n = 5/group). Similar results were obtained in the flow cytometry analysis of LLN cells. The number of IL-4 secreting CD4^+^ T cells was found to be significantly increased (Figure 7 and Supplementary Figure S2, p < 0.05, AE vs. PBS, n = 5/group). Furthermore, mean fluorescence intensity of the IL-4^+^ CD4^+^ cells was significantly increased in the AE-treated group compared with the control group (Figure 7, right panel).

## 4. Discussion

*Anisakis* is a marine parasite that infects humans due to the consumption of marine fish. In 2011, 400 infections were reported in South Korea, since which the number of infections has steadily risen to 800 in 2018 [47–49]. *A. simplex* has been reported to act as an allergen that causes asthma in workers handling fishes [7–9, 50]. *A. pegreffii* is a dominant marine parasitic species, compared with other *Anisakis* species, causing infections in the South Korean population [51, 52]. Furthermore, *A. pegreffii* may induce allergies as well based on the presence of *A. pegreffii*-tropomyosin in the serum of patients with crustacean allergy [53]. *A. pegreffii*-induced allergies have also been reported in animal models [13, 35, 54]. *A. simplex* and *A. pegreffii* have been reported as different species with genetic differences [55–57] but can be distinguished only at the L4 or adult stages [58]. Allergens of Ani pe 1, 2, 12, and 13 have already been reported for *A. pegreffii* [17]. Furthermore, it was reported that it is possible to detect allergens sensitive to IgE in the sera of *Anisakis*-infected patients. This indicates that these two species, although with slight genetic differences, can cause the “*Anisakis* allergy” [59].

To date, there is a lack of model systems that replicate the airway remodeling properties of asthma among animal models using *Anisakis*. In the present study, the phenotypic characteristics of allergic airway inflammation were compared using a protocol comprising chronic 4-week exposure to the AE antigen associated with the allergen. The 4-week AE model using daily AE exposure for 5 days a week showed enhanced MCh reactivity in AHR, similar to the 4-week house dust mite (HDM) model [43]. Airway inflammation, as measured by leukocyte recruitment to the airway, was significantly greater in the AE treatment group than in the control group. Similar to that in the 4-week HDM model [43], leukocytosis in BALF associated with the 4-week AE model was primarily due to an increase in the eosinophil and neutrophil population. Our observations for the 4-week AE group were similar to those from the previously reported 4-week HDM model [43], with a substantial increase in eosinophils accompanied by a significant increase (39.45%) in the number of BAL monocytes along with the total absolute monocyte count.

We also observed a greater extent of allergic reactions (e.g., HDM-specific IgE, IgG1, and IgG2a levels) in the AE 4-week model than in the control group (Figure 3). Several animal allergic disease models have been developed to elucidate the immune mechanism of anisakiasis. Specifically, IgE, IgG1, and at times IgG2a were detected in the serum after an HDM, *Trichinella spiralis*, and ovalbumin (OVA) infection [60–63]. Moreover, Th2 cytokines were produced in some cases, even if a mixed Th1/Th2 cell response was observed [29, 64–66]. Allergic reactions to *A. simplex* induce a type I hypersensitivity reaction in guinea pigs, rabbits, and humans, and anti-*A. simplex* IgE levels increase rapidly at the beginning of infection [67]. Furthermore, in an infection model in which *A. pegreffii* was administered orally after intraperitoneal infection, the concentrations of IgE, IgG1, and IgG2a in the blood steadily increased up to 4 weeks after infection [66]. Thus, the results of our study are similar to previous findings. Continuous intranasal challenge of AE for 4 weeks suggested that this method was suitable for inducing allergic reactions in mice (Figure 3).

With regard to the allergic airway remodeling in the AE-treated mice, an increased number of PAS^+^ cells were observed 4 weeks after AE sensitization/exposure (Figure 5(b)). Masson's trichrome staining of the airways confirmed peribronchial collagen deposition in the 4-week AE model (Figure 5(a)). Our findings were similar to those of a previous study wherein the main features of airway remodeling, namely, epithelial thickening, smooth muscle proliferation, and collagen deposition, required sustained exposure to HDM allergens for at least 4 weeks [43].

Our investigation of the inflammatory response demonstrated differences in the leukocyte recruitment mediator gene expression compared with HDM-induced asthma. The neutrophil mediators Cxcl-1 and IL-17A are expressed at the onset of neutrophil inflammation and have been reported to show the highest levels in a 2-week HDM exposure model [33]. In contrast, in our 4-week AE model, there was no change or a tendency to decrease when compared with the PBS control group (Supplemental Figure S1). We also observed increased expression of IL-6 and eotaxin-1 in the 4-week AE model; however, eosinophil recruitment to the airways was not significant despite an increase of 13.07% compared with that in the PBS control. These differences in observations may be due to differences in the number, frequency, and duration of allergen exposure in the protocols used in the two studies, as well as the specific allergens used.

A hallmark of asthma is elevation in the levels of Th2-type cytokines, such as IL-4 and IL-13 [31, 33, 67]. We observed an increasing trend in *IL-4* and *IL-13* mRNA expression in the 4-week model. Indeed, increased levels of the IL-4 and IL-13 cytokines in the mouse *A. pegreffii* model have previously been reported [7]. IL-6 is a cytokine that controls the pathogenesis of asthma and the early stages of development of Th2 cells, a biomarker of asthma exacerbation [68]. In our model, the expression of IL-6 increased at 4 weeks of *A. pegreffii* exposure, consistent with the maintenance of airway remodeling. Although previous studies have demonstrated AHR of *A. pegreffii*-induced allergic airway inflammation, they found that it showed airway inflammation, systemic allergic reaction, airway remodeling, and other phenotypic features in other *A. pegreffii* mouse models [69, 70]. These differences may be related to changes in the frequency, dosage, and duration of allergen exposure. Our 4-week model revealed that the expression of neutrophil inflammation and associated mediators, such as Cxcl-1 and IL-17A, did not trigger an initial inflammatory response compared to that in the PBS control group. We detected an increase in *eotaxin-1* gene expression; however, the increase was not statistically significant. In 2 weeks, the model using *A. pegreffii* allergen appeared to similarly exhibit acute allergic airway inflammation.

Thus, the chronic allergic airway inflammation model described in this study potently recapitulated airway remodeling with collagen deposition.

### 4.1. Conclusion Remarks and Perspectives

We propose that our 4-week 25 *μ*g AE model may be a suitable method for studying asthma characterized by AHR, airway inflammation, and airway remodeling through collagen deposition. A limitation of our experiment was that asthma symptoms were reproduced with an intranasal challenge rather than as droplets (asthma symptoms caused by *Anisakis* in workers are mainly due to droplets). Another limitation is that we did not attempt to determine a therapeutic agent to treat asthma accompanying the airway remodeling phenomenon induced in mice. Future experiments should explore these avenues further and reveal the correlation with intestinal bacteria when *Anisakis*-induced asthma develops.

## Data Availability

The authors can provide the data analyzed in this study upon reasonable request.

## Supplementary Materials

Supplementary Materials Supplementary Figure S1: expression of eotaxin-1, IL-6, and IFN-*γ* is upregulated with AE exposure. Expression of eotaxin-1, IL-6, and IFN-*γ* (A, D, E, F) was upregulated in the AE model compared with the PBS control group. However, Cxcl1 and IL-17a expression did not differ from that of the control group (B, C) (^∗^*p* < 0.05; *n* = 5/group). Supplementary Figure S2: expression of the IL-4^+^ CD4^+^ marker in splenocytes. Expression of the IL-4 marker in the splenocytes of AE-sensitized and control mice was analyzed using flow cytometry. Lymphocytes from splenocytes were incubated with a stimulated anti-CD3e antibody. After staining, lymphocytes were initially gated for CD4^+^ cells, and the percentage of IL-4^+^ cells was calculated using FACS analysis. The IL-4^+^ CD4^+^ T cell number is plotted in the right panel. Supplementary Table 1: primer sequence, target gene, and cycling conditions for SYBR green RT-PCR.
