# Supplementary material for: Anisakis pegreffii Extract Induces Airway Inflammation with Airway Remodeling in a Murine Model System
Source: Biomed Res Int. 2021 Sep 17;2021:2522305. doi: 10.1155/2021/2522305 (PMC8464433; doi:10.1155/2021/2522305)
Supplement: Supplementary Materials — Supplementary Figure S1: expression of eotaxin-1, IL-6, and IFN-γ is upregulated with AE exposure. Expression of eotaxin-1, IL-6, and IFN-γ (A, D, E, F) was upregulated in the AE model compared with the PBS control group. However, Cxcl1 and IL-17a expression did not differ from that of the control group (B, C) (∗p < 0.05; n = 5/group). Supplementary Figure S2: expression of the IL-4+ CD4+ marker in splenocytes. Expression of the IL-4 marker in the splenocytes of AE-sensitized and control mice was analyzed using flow cytometry. Lymphocytes from splenocytes were incubated with a stimulated anti-CD3e antibody. After staining, lymphocytes were initially gated for CD4+ cells, and the percentage of IL-4+ cells was calculated using FACS analysis. The IL-4+ CD4+ T cell number is plotted in the right panel. Supplementary Table 1: primer sequence, target gene, and cycling conditions for SYBR green RT-PCR. [file 2522305.f1.zip › Supplementary Figure S1_210824.pptx]

## Slide 1
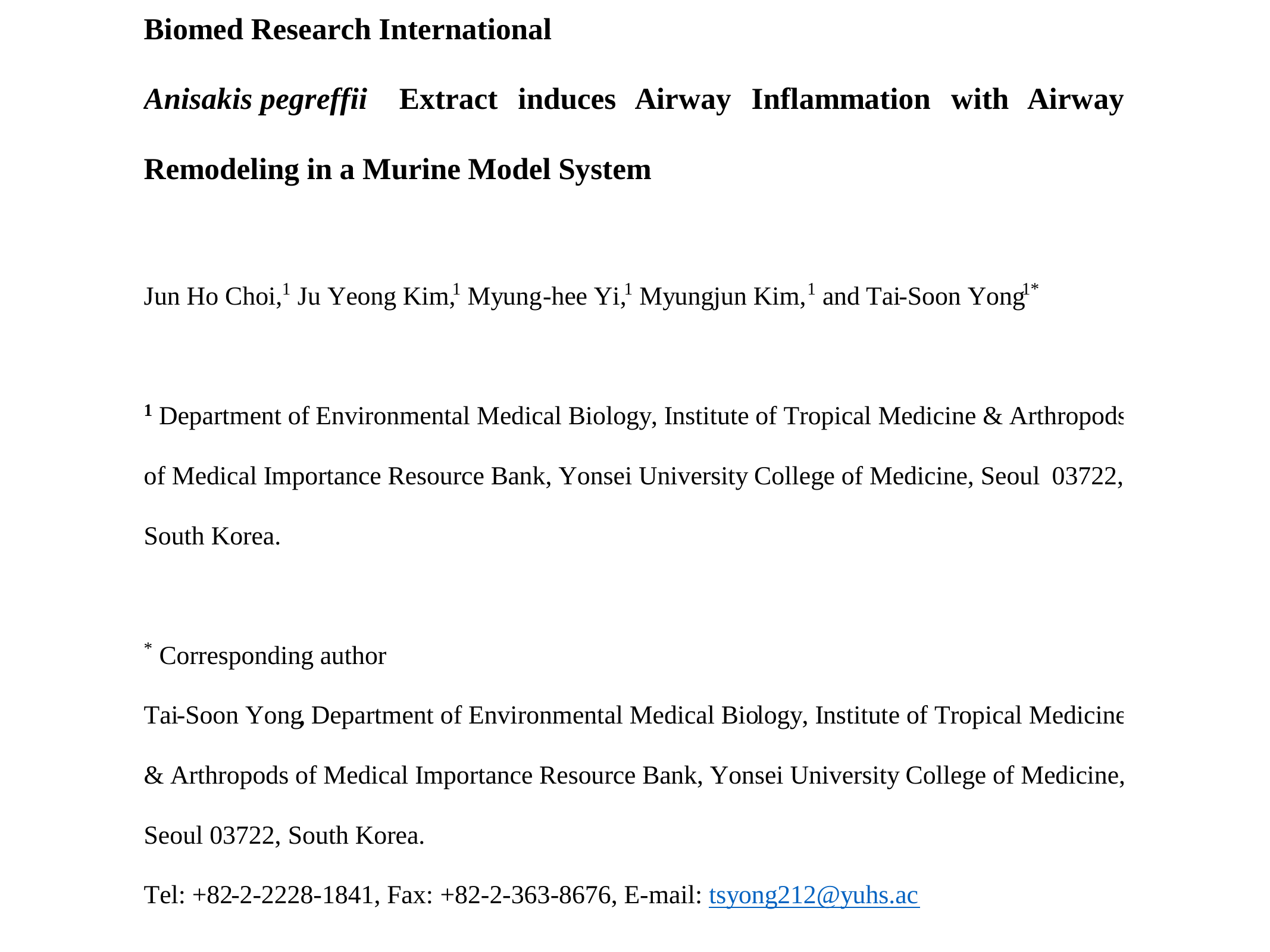

1

## Slide 2
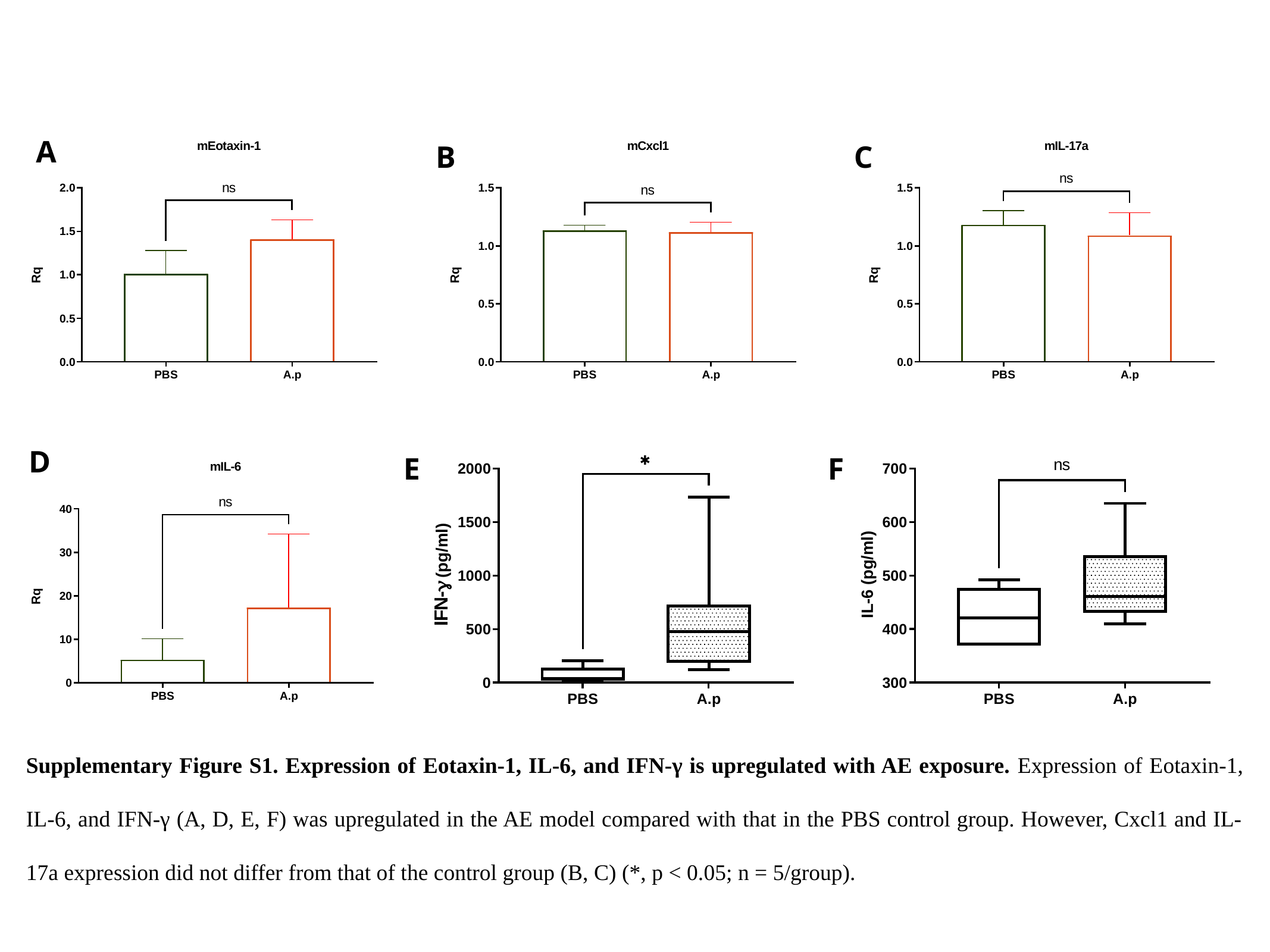

A
B
C
D
E
F
Supplementary Figure S1. Expression of Eotaxin-1, IL-6, and IFN-γ is upregulated with AE exposure. Expression of Eotaxin-1, IL-6, and IFN-γ (A, D, E, F) was upregulated in the AE model compared with that in the PBS control group. However, Cxcl1 and IL-17a expression did not differ from that of the control group (B, C) (*, p < 0.05; n = 5/group).
