# Supplementary material for: Anisakis pegreffii Extract Induces Airway Inflammation with Airway Remodeling in a Murine Model System
Source: Biomed Res Int. 2021 Sep 17;2021:2522305. doi: 10.1155/2021/2522305 (PMC8464433; doi:10.1155/2021/2522305)
Supplement: Supplementary Materials — Supplementary Figure S1: expression of eotaxin-1, IL-6, and IFN-γ is upregulated with AE exposure. Expression of eotaxin-1, IL-6, and IFN-γ (A, D, E, F) was upregulated in the AE model compared with the PBS control group. However, Cxcl1 and IL-17a expression did not differ from that of the control group (B, C) (∗p < 0.05; n = 5/group). Supplementary Figure S2: expression of the IL-4+ CD4+ marker in splenocytes. Expression of the IL-4 marker in the splenocytes of AE-sensitized and control mice was analyzed using flow cytometry. Lymphocytes from splenocytes were incubated with a stimulated anti-CD3e antibody. After staining, lymphocytes were initially gated for CD4+ cells, and the percentage of IL-4+ cells was calculated using FACS analysis. The IL-4+ CD4+ T cell number is plotted in the right panel. Supplementary Table 1: primer sequence, target gene, and cycling conditions for SYBR green RT-PCR. [file 2522305.f1.zip › Supplementary Figure S2_210824.pdf]

Biomed Research International

Anisakis pegreffii Extract induces Airway Inflammation with Airway Remodeling in a Murine Model System

Jun Ho Choi,[1] Ju Yeong Kim,[1] Myung-hee Yi,[1] Myungjun Kim,[1] and Tai-Soon Yong[1*]

[1] Department of Environmental Medical Biology, Institute of Tropical Medicine & Arthropods of Medical Importance Resource Bank, Yonsei University College of Medicine, Seoul 03722, South Korea.

[*] Corresponding author

Tai-Soon Yong Department of Environmental Medical Biology, Institute of Tropical Medicine & Arthropods of Medical Importance Resource Bank, Yonsei University College of Medicine, Seoul 03722, South Korea.

Tel: +82-2-2228-1841, Fax: +82-2-363-8676, E-mail: tsyong212@yuhs.ac

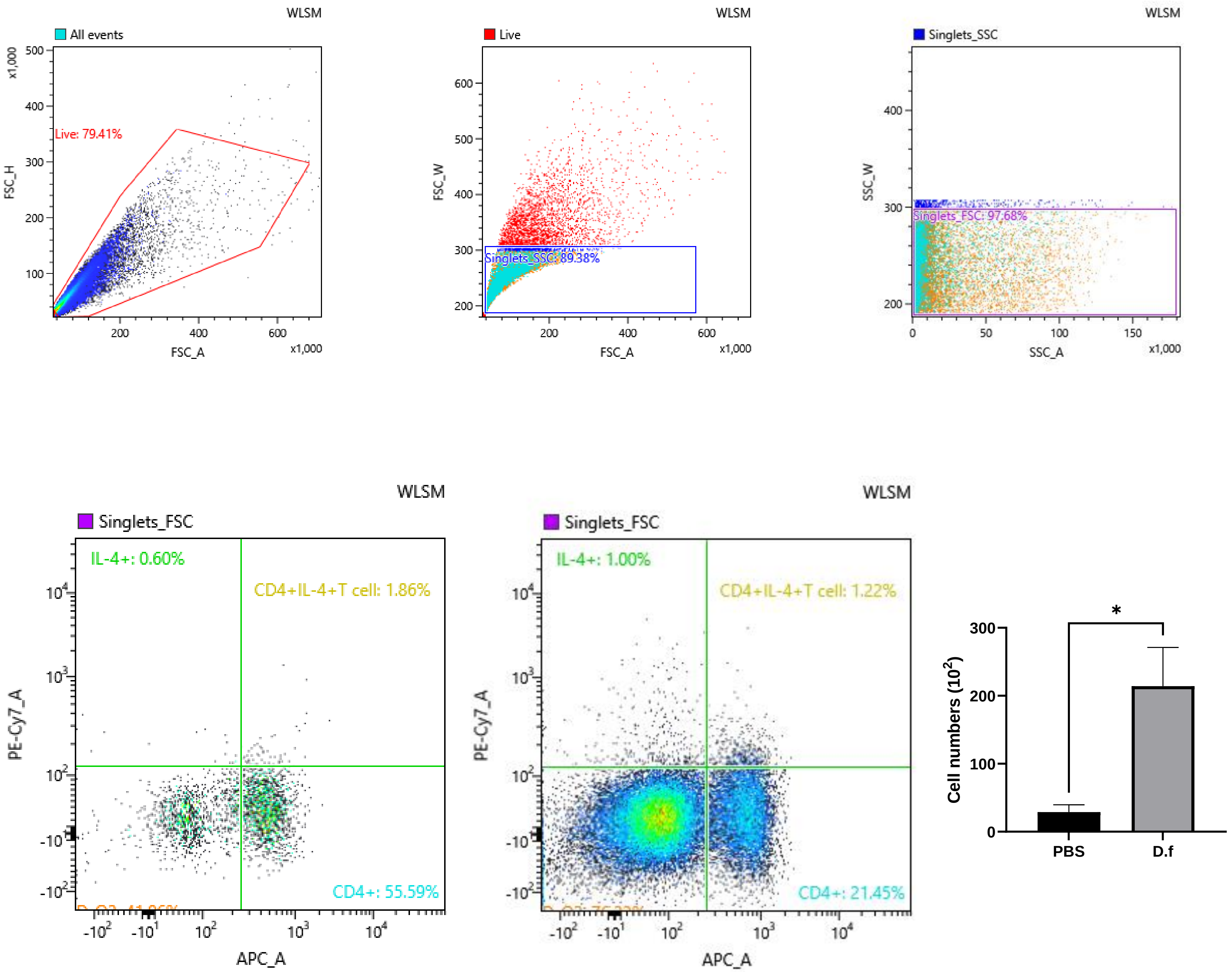

Supplementary Figure S2. Expression of the IL-4$^+$ CD4$^+$ marker in splenocytes. Expression of the IL-4 marker in the splenocytes of AE-sensitized and control mice was analyzed using flow cytometry. Lymphocytes from splenocytes were incubated with a stimulated anti-CD3e antibody. After staining, lymphocytes were initially gated for CD4$^+$ cells, and the percentage of IL-4$^+$ cells was calculated using FACS analysis. The IL-4$^+$ CD4$^+$ T cell number is plotted on the right panel.
