# Supplementary material for: Anisakis pegreffii Extract Induces Airway Inflammation with Airway Remodeling in a Murine Model System
Source: Biomed Res Int. 2021 Sep 17;2021:2522305. doi: 10.1155/2021/2522305 (PMC8464433; doi:10.1155/2021/2522305)
Supplement: Supplementary Materials — Supplementary Figure S1: expression of eotaxin-1, IL-6, and IFN-γ is upregulated with AE exposure. Expression of eotaxin-1, IL-6, and IFN-γ (A, D, E, F) was upregulated in the AE model compared with the PBS control group. However, Cxcl1 and IL-17a expression did not differ from that of the control group (B, C) (∗p < 0.05; n = 5/group). Supplementary Figure S2: expression of the IL-4+ CD4+ marker in splenocytes. Expression of the IL-4 marker in the splenocytes of AE-sensitized and control mice was analyzed using flow cytometry. Lymphocytes from splenocytes were incubated with a stimulated anti-CD3e antibody. After staining, lymphocytes were initially gated for CD4+ cells, and the percentage of IL-4+ cells was calculated using FACS analysis. The IL-4+ CD4+ T cell number is plotted in the right panel. Supplementary Table 1: primer sequence, target gene, and cycling conditions for SYBR green RT-PCR. [file 2522305.f1.zip › Supplementary Table 1-BioMed_Research_International_210824.docx]

Biomed Research International

*Anisakis pegreffii* Extract induces Airway Inflammation with Airway Remodeling in a Murine Model System

Jun Ho Choi,^1^ Ju Yeong Kim,^1^ Myung-hee Yi,^1^ Myungjun Kim,^1^ and Tai-Soon Yong^1*^

**^1^** Department of Environmental Medical Biology, Institute of Tropical Medicine & Arthropods of Medical Importance Resource Bank, Yonsei University College of Medicine, Seoul 03722, South Korea.

^*^ Corresponding author

Tai-Soon Yong, Department of Environmental Medical Biology, Institute of Tropical Medicine & Arthropods of Medical Importance Resource Bank, Yonsei University College of Medicine, Seoul 03722, South Korea.

Tel: +82-2-2228-1841, Fax: +82-2-363-8676, E-mail: [tsyong212@yuhs.ac](mailto:tsyong212@yuhs.ac)

Supplementary Table 1: Primer sequence, target gene, and cycling conditions for SYBR green RT-PCR

| Target gene | Primers sequences | Reverse transcription | Primary  Denaturation | Amplification (40 cycles) | | Dissociation curve (1 cycle) | | | References |
| --- | --- | --- | --- | --- | --- | --- | --- | --- | --- |
|  |  |  |  | Secondary denaturation | Annealing | Secondary denaturation | Annealing | Final denaturation |  |
| Ppia | ACCAAACACAAACGGTTCCCA | 42 °C  30 min | 95 °C  10 min | 95 °C  15 sec | 54.5 °C  1 min | 95 °C  15 sec | 54.5 °C  1 min | 95 °C  15 sec | [43] |
|  | GCTTCCACAATGTTCATGCCTT |  |  |  |  |  |  |  |  |
| IL-4 | ATGTACCAGGAGCCATATCCAC |  |  |  | 56.5 °C  1 min |  | 56.5 °C  1 min |  |  |
|  | GGCTCAGTACTACGAGTAATCCA |  |  |  |  |  |  |  |  |
| Eotaxin-1  (chemokine, C-C motif) ligand 11, Ccl11) | ACAGATGCACCCTGAAAGCCAT |  |  |  | 57.7 °C  1 min |  | 57.7 °C  1 min |  |  |
|  | GGCGACTGGTGCTGATATTCCCT |  |  |  |  |  |  |  |  |
| IL-5 | GTGGGGGTACTGTGGAAATG |  |  |  | 60.0 °C  30 sec |  | 60.0 °C  30 sec |  | [44] |
|  | TCTCTCCTCGCCACACTTCT |  |  |  |  |  |  |  |  |
| IL-13 | AGACCAGACTCCCCTGTGCA |  |  |  | 58.0 °C  1 min |  | 58.0 °C  1 min |  | [45] |
|  | TGGGTCCTGTAGATGGCATTG |  |  |  |  |  |  |  |  |
| IL-17A | CCTCAGACTACCTCAACCGTTC |  |  |  | 57.6 °C  1 min |  | 57.6 °C  1 min |  | [43] |
|  | AGCTTTCCCTCCGCATTGACA |  |  |  |  |  |  |  |  |
| Cxcl1 | ACCCAAACCGAAGTCATAGCC |  |  |  |  |  |  |  |  |
|  | GTCAGAAGCCAGCGTTCACCA |  |  |  |  |  |  |  |  |
